# Transcriptional Networks in Epithelial-Mesenchymal Transition

**DOI:** 10.1371/journal.pone.0025354

**Published:** 2011-09-30

**Authors:** Christo Venkov, David Plieth, Terri Ni, Amitava Karmaker, Aihua Bian, Alfred L. George, Eric G. Neilson

**Affiliations:** 1 Department of Medicine, Vanderbilt University School of Medicine, Nashville, Tennessee, United States of America; 2 Department of Cell Biology, Vanderbilt University School of Medicine, Nashville, Tennessee, United States of America; 3 Department of Biostatistics, Vanderbilt University School of Medicine, Nashville, Tennessee, United States of America; 4 University of Wisconsin-Stout, Menomonie, Wisconsin, United States of America; City of Hope National Medical Center and Beckman Research Institute, United States of America

## Abstract

**Backround:**

Epithelial-mesenchymal transition (EMT) changes polarized epithelial cells into migratory phenotypes associated with loss of cell-cell adhesion molecules and cytoskeletal rearrangements. This form of plasticity is seen in mesodermal development, fibroblast formation, and cancer metastasis.

**Methods and Findings:**

Here we identify prominent transcriptional networks active during three time points of this transitional process, as epithelial cells become fibroblasts. DNA microarray in cultured epithelia undergoing EMT, validated *in vivo*, were used to detect various patterns of gene expression. In particular, the promoter sequences of differentially expressed genes and their transcription factors were analyzed to identify potential binding sites and partners. The four most frequent *cis*-regulatory elements (*CREs*) in up-regulated genes were *SRY*, *FTS-1*, *Evi-1*, and *GC-Box*, and RNA inhibition of the four transcription factors, *Atf2, Klf10, Sox11, and SP1*, most frequently binding these *CREs*, establish their importance in the initiation and propagation of EMT. Oligonucleotides that block the most frequent *CREs* restrain EMT at early and intermediate stages through apoptosis of the cells.

**Conclusions:**

Our results identify new transcriptional interactions with high frequency *CREs* that modulate the stability of cellular plasticity, and may serve as targets for modulating these transitional states in fibroblasts.

## Introduction

Epithelial-mesenchymal transition (EMT) is a classic mechanism of cellular plasticity [1], [2]. EMT events fall into three subtypes based on context: Type 1, involving primitive epithelial cells transitioning to mesenchymal cells during embryological formation of the early body plan [3], [4]; Type 2, involving secondary epithelial [5], [6] or endothelial [7], [8] cells transitioning to tissue fibroblasts during fibrogenesis, and; Type 3, involving epithelial carcinoma cells in primary nodules that transition to a metastatic phenotype [9], [10]. The subject of EMT in the kidney has been reviewed recently [6], [11], [12].

Initiation of EMT in fibrosis is dependent on outside-in signaling by transforming growth factor-β (TGF-β) [13], [14] as well as other effector molecules including ILK, EGF, and FGF-2 [6]. EMT transition results in the up-regulation of transcriptional modulators such as CBF-A [15], Snail [16], Twist [17], HMG A2 [18], β-catenin [19], LEF 1, Zeb2/Sip1 [20] and Smad [21], the loss of adherence molecules, and the gain of new moieties important for cell movement [1], [9]. The direct overexpression of *snail* [22] or *twist* [23] classically induce EMT and fibrosis in adult tissues, and the binding of CBF-A to the *FTS-1 cis*-regulatory element *(CRE)* in the promoters of some of the prototypical EMT genes serves as a key modulating event [15].

Sequence-specific binding of transcription factors is prerequisite for transcriptional regulation through gene regulatory networks [24], [25], [26], [27], [28] and *CREs* are essential for negotiating specific recognition between regulatory factors and nucleotide sequences [29], [30]. Still, there is no clear pattern recognition for the regulatory networks in the initiation and maintenance of the molecular program of EMT. Here we characterize the gene expression patterns of epithelia at different stages of transition by DNA microarray using proximal tubular epithelial cells induced with cytokines [14] and validated *in vivo* in a model of kidney fibrosis [11]. Our results suggest that networks of *CREs* define the binding of preferred transcription factors and their complexes to drive pivotal genes in the EMT program.

## Materials and Methods

### Cell cultures and stimulation

The kidney proximal tubular cell line, MCT, was derived from mice [31]. For initiation of EMT, epithelial cells were fed with a low-serum medium overnight, and induced with 3 ng/ml TGF-β1 and 10 ng/ml EGF as described elsewhere [13], [14]. NIH/3T3 were used as a control fibroblast cell line.

### Unilateral urethral obstruction (UUO)

We followed the procedure as described earlier [15]. The Institutional Animal Care and Use Committee (IACUC) at Vanderbilt University approved all animal studies. Three-month-old male Balb/c mice (Jackson Laboratories, Bar Harbor, ME) were used and mice were monitored postoperatively. Both fibrotic and contralateral kidneys were harvested at several time points up to 7 days after surgery and snap-frozen in liquid nitrogen. RNA was extracted from crushed frozen tissue by homogenization in Trizol (Invitrogen, Carlsbad, CA) according to manufacturer's instructions. The expression pattern of selected genes was monitored by qRT-PCR as described below.

### Microarray analysis

Total RNA was collected from the cells at different time points using Trizol (Invitrogen), converted to cDNA, and hybridized to microarray slides using the Affymetrix Mouse GeneChip Mu-430A Oligo Set (Affymetrix, Santa Clara, CA) at the Vanderbilt University Microarray facility. To identify the common regulated genes between transitioning epithelia and the fibroblast endpoint, the pattern of differential expression at a given time point (time point N versus time point 0), was compared with that of 3T3 fibroblasts versus time point 0 (non-induced epithelium). Data were collected from three separate experiments for each time point and subjected to analyses to satisfy criteria for statistical significance and cutoff.

### Data normalization, scoring, and analysis

Briefly, raw intensity data were background-subtracted using maximum likelihood estimation and normalized to the baseline array by two-dimensional variable reduction and approximation (NVRA) method to remove systematic nonlinear noise between paired arrays [32]. Sample sizes for microarray experiments are generally small and may vary among treatment groups. We thus used a rank-based differential expression method of analysis, which takes advantages of unique multiple measurements per transcript design implemented in Affymetrix chip arrays (see Methods S1 for details). An absolute fold change of 1.75 was used to select genes with differential expression. Venn diagrams were compiled using an online tool (www.SmartDraw.com). Differentially expressed genes were grouped according to their quintessential functions using the Excel bioinformatics tool (http://www.helsinki.fi/project/ritvos/GoCore/). Functional clustering of differentially expressed genes was performed using DAVID Bioinformatic Resources (NIAID, NIH). Pathway maps were derived as follows: the differentially expressed genes and their log2 expression ratios between untreated versus treated were imported into PathwayAssist software (Ariadne Genomics, Rockville, MD) using gene IDs supplied by Affymetrix annotation files. Pathways were constructed using find only direct interactions between selected entities option.

### siRNA inhibition

Three pre-designed Stealth Select siRNAs™ (Invitrogen, Carlsbad, CA) for each targeted gene, were used for transfection using Lipofectamine 2000 (Invitrogen) and pre-tested for levels of RNAi by quantitative real-time PCR (qRT-PCR). The most effective siRNAs were adopted for the study together with a specificity control siRNA that did not suppress the target. The levels of transcriptional repression were monitored by qRT-PCR using D-Lux primers/probes (Invitrogen) at different time points after transfection. For monitoring the effect of RNAi on the process of EMT, parallel MCT cultures were transfected with siRNA targeting a particular transcript or its specificity control and 24 hours later one of the cultures was induced for EMT (see above). RNA was extracted 6 or 18 hours after induction for qRT-PCR analysis. In addition, separate cultures treated similarly, were used as a source of total protein for Western blot analysis 18 hours after EMT induction.

### Western blot analysis

Blots were performed as previously described [15]. Our previous experiments showed at different stages of EMT that the expression of several housekeeping genes commonly used for normalization, including GPDH and β-actin, vary over time as described by others [33] and their use is not applicable [15]. Instead we used Coomassie blue staining of replicate gels as loading controls. In preliminary experiments we stained membranes after immunoblotting, before immunoblotting, or the gels after transfer [34], [35]. There were no substantial quantitative differences between the stained protein bands using either of these techniques. For this reason, the loading controls in the Western blots here represent staining of replicate gels.

### qRT-PCR analysis

Extracted total RNA from cell cultures was treated with DNAse I, and converted by reverse transcription to single-stranded cDNA using Superscript reverse transcriptase (Invitrogen, CA). Primers and FAM labeled probes for the EMT marker genes were from TaqMan Gene Expression Assays (Applied Biosystems, CA). The primers and D-Lux probes for the siRNA- targeted transcription factors were from Invitrogen. Quantitative PCR reactions were performed in 96-well format using an Applied Biosystems 7900HT Fast PCR System and Taqman Fast Universal Master Mix (Applied Biosystems).

All qRT-PCR experiments were performed three times as separate experiments and each biological replicate in each experiment was run in triplicate, according to standard protocol [15]. Data expressed as cycle threshold (Ct) values were normalized to the amount of input cDNA and calculated as fold change in comparison to control values using the Excel bioinformatics tool and the formula [ = power(2,-(xn-yn))], where xn and yn are the positions of treatment and control. Data are presented as means and standard deviations. Previous experiments demonstrated that the expression of several common housekeeping genes used for normalization change at different stages of EMT and are not useful in this setting. Instead we adopted normalization for qRT-PCR samples based on their optical density at 260 nm, which we [15] and others [36] have used successfully. Normalization based on the total cellular RNA content is increasingly used, as total cellular RNA is the ‘least unreliable’ method [37], [38]. Equal amounts of RNA was used for the RT reactions, the cDNA generated was again quantitated by UV, diluted to equal absorbance as working samples, and then again screened by UV to account for possible differences in dilution.

For experiments using cells transfected with CRE decoys, a one-way ANOVA was used to assess the overall level of significance across experimental inhibitors and control inhibitor for each time point separately. The post hoc Bonferroni-corrected t-test was used to compare mean differences between each of 4 experimental inhibitors (siRNA or decoys) and their relevant controls whereby ANOVA detects overall level of significance. All data analyses were performed using SAS 9.1.3 (SAS Institute, Cary, NC); a significance level of 0.05 was used for statistical inferences.

Results from experiments with cells transfected with siRNA for the 4 transcription factors were used to compile the transcriptional network relationships presented in the text. For statistical evaluation we used Student's *t*-test with a Bonferroni adjustment post hoc to assess differences between groups. Statistical analyses were performed using R version 2.10.0 (http://www.r-project.org) and 2-sided P values less than 0.05 were considered statistically significant.

### Identification and charting of cis-regulatory elements

For each time point (T6, T18 and T96), we selected groups of up-regulated (expression value greater than 1.75) and down-regulated (expression value less than −1.75) genes, then performed a sequence of three analysis steps. For each gene, the sequence of the promoter region was delineated as 1500 bp upstream to 500 bp downstream (−1500 to +500, total 2000 bps) of the annotated TSS. Promoter sequences were annotated as FASTA format and fed to TFM-Explorer [39], which interfaces with TRANSFAC [40] to identify putative *cis*-regulatory elements along the promoter sequences. TRANSFAC, the largest repository of experimentally validated transcription factor binding sites, predicted CREs in the sequence limits mentioned based on a start site of 0. The total counts for individual *cis*-elements were tabulated for each gene group and then *cis*-regulatory elements were sorted based on their frequencies across all time points. With the top six most frequent, common *cis*-elements in each gene group (up- or down-regulated), total numbers of their occurrences were plotted at different time points. In the plot, the total counts of respective *cis*-elements were shown as percentile of their frequency in the total number of genes selected at that time point.

### Oligonucleotide decoys (Dumbbells)

Concatenated sequences with repeats identical to the four most frequent *cis*-elements were synthesized (IDT, Coralville, IA) and ligated as described [41]. They were transfected into MCT cells as described above for siRNA.

*Evi-1:* ODN1: 5′-tttatcttggctgtttaccttgtctgcttggtttttccaag, ODN2: 5′-cagacaaggtaaacagccaagat aaaccgtctttttgacgg; *FTS-1:* ODN1: 5′-tgattgatccttgattgatcctcttggtttttccaag, ODN2: 5′-aggatcaatc aaggatcaatcaccgtctttttgacgg; *SRY:* ODN1: 5′-acttttgttttttacttttgttttttcttggtttttccaag, ODN2: 5′-aaaaaacaaaagtaaaaaacaaaagtccgtctttttgacgg; *GC-Box:* ODN1: 5′-ggccccgccccggtgccccgcccct gcttggtttttccaag, ODN2: 5′-accggggcggggccaccggggcggggccccgtctttttgacgg; *DB-control:* ODN1: 5′- actgactgactgactgactgactgcttggtttttccaag, ODN2: 5′- cagtcagtcagtcagtcagtcagtccgtctttttgacgg.

Apoptosis of cells transfected with the oligonucleotide decoys was evaluated by the In Situ cell Death Detection kit (Roche Diagnostics, Mannheim, Germany) according to manufacturer's instructions.

### EMSA

EMSA was performed as described previously [15]. Nuclei were purified from cultured cells using the Nuclei EZ Prep isolation kit (Sigma-Aldrich, St. Louis, MO) according to manufacturer's instructions. Nuclear extracts were prepared in 400 mM KCl buffered with 20 mM HEPES pH 7.9 supplemented with a cocktail of proteinase inhibitors (Sigma-Aldrich). The protein extracts were brought down to 100 mM KCl by dialysis and the protein content of the supernatant was estimated using the DC Protein assay (BioRad, Hercules, CA). For competition analyses, protein was pre-incubated with unlabeled oligonucleotides.

## Results

### Network patterns of transcription during EMT

**Figure 1:**
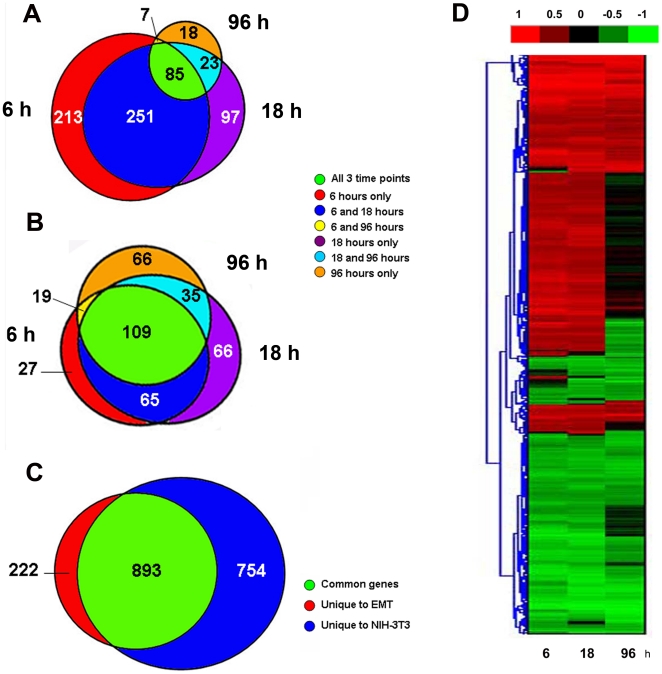
Distribution and clustering of differentially expressed genes during EMT progression. (A–C) Venn diagrams. The number of genes changed at each stage is indicated and the various stages are colored as follows: red- 6 hours; violet-18 hours and yellow- 96 hours. (**A**) Up-regulated, (**B**) Down-regulated. Labeled with green are genes shared by all three stages; genes shared between two stages are labeled as shown. (**C**) Genes with changed expression in EMT and in NIH 3T3 fibroblasts in comparison to naïve MCT. (**D**). Hierarchical clustering of the genes changed at various stages of EMT. Red- up-regulated, green- down-regulated; black- not changed. The color bar at the top displays the levels of differential expression based on their relative expression levels and statistical significance (see Methods).

The transcriptomes of tubular epithelial cells undergoing EMT to form fibroblasts and of control fibroblasts relative to non-induced epithelium were interrogated by DNA microarray at three time points: early (6 hours), intermediate (18 hours) and late (96 hours) [14]. Samples after 48 hours of EMT were also investigated by DNA microarray and showed that the gene expression pattern is intermediate between 18 and 96 hours (data not shown). EMT is a continuum and there is no definitive timepoint at which drastic deviations occur. Divergence in the gene expression pattern is observed as early as 6 hours after induction and morphological changes appear gradually over several days. After 18–24 hours, cells start to assume a more ellipsoidal appearance and detach from sister cells. Their numbers gradually increase and at 96 hours the fibroblast morphology totally prevails. Venn diagrams of up or down-regulated genes at each time point illustrate the number of genes unique or shared between time points (Figure 1). The number of up-regulated genes (A) during the first 6 to 18 hours (dark blue) is considerably higher than down-regulated genes (B) from the same time period. More than 60% of all differentially expressed genes are up-regulated (Table S1), with the highest percentage (72%) in the first 6 hours maintained through 18 hours (63%). This percentage strikingly declines to 35% at 96 hours, which marks the end of the transition to fibroblasts [15].

The transcriptome of newly forming fibroblasts compared to control fibroblasts (Figure 1C) share a large number of differentially expressed genes (893) that represent 80% of all changed genes and 54% of the genes found in fibroblasts. Figure 1D illustrates the hierarchical clustering of EMT genes using a scale normalized by non-parametric variable reduction and approximation for differentially expressed genes that are statistically different from non-induced controls (P<0.01) [32]. This hierarchical clustering aggregates the changes at various stages of EMT with the majority of up-regulated genes (red) during the first 18 hours of induction and a noticeable increase in down-regulated (green) or unchanged (black) genes at 96 hours.

### Validation of expected EMT markers in cells and a model of tissue fibrosis

**Figure 2:**
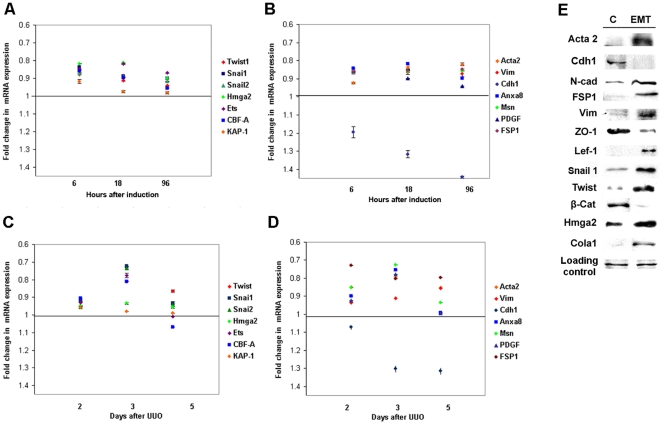
The EMT marker genes are also differentially expressed when monitored by qRT-PCR and immunoblotting. (**A, B**) in MCT cells induced to EMT; (**C, D**) in the fibrotic kidney. The mean results from three independent replicates are expressed as fold change compared to the expression in non-induced naïve MCT cells. (**E**). Immunoblotting of total protein extracted from MCT cells induced to EMT after 18 hours, or naïve controls (**C**). The loading controls for the Western represent Coomassie staining of replicate gels as the expression of housekeeping genes is not stable throughout EMT.

We employed a classical model of kidney fibrosis following urethral obstruction [1]. Based on the EMT literature [3], [15], [21], [42], [43], we also selected marker genes (transcription factors: *Snail1*, *Snail 2*, *Twist 1*, *Hmga2*, *CBF-A*, *KAP-1*, and *Ets* and non-transcription factors: *FSP1*, *moesin, annexin 8, PDGF*, *vimentin, αSMA*, and *E-cadherin*) to monitor transition using both qRT-PCR and protein expression [15], [43] . The test samples used to generate the microarray data were probed for mRNA expression (Figure 2A,C) and protein for immunoblotting was obtained at 18 hours after induction of EMT (Figure 2E). The results confirm expected differential expression of EMT markers. The relevance of the cell culture system was also validated by monitoring the expression of the same gene set in mouse kidneys with fibrosis (Figure 2, C and D), where most interstitial fibroblasts derive from EMT [Iwano, 2002 #35;Zeisberg, 2010 #472;Zeisberg, 2010 #439]. The similar expression pattern of expected EMT markers *in vitro* and *in vivo* confirms their relevance to transition from epithelial cells to fibroblasts.

### Organization of the molecular networks encoding EMT

Hierarchical clusters of EMT genes were grouped according to their known functions using the DAVID Bioinformatics Resource (NIAID) and Pathway Assistance Software (Ariadne Genomics). The most populated clusters include genes involved with the cell cycle, cell adhesion, endoplasmic reticulum, extracellular matrix, transcription factor, inflammation, and cytoskeleton (Figure S1). The predominant functions in each cluster at each stage of the transition are defined in Table S2. The major networks activated in EMT at early and intermediate stages are related to the cellular inflammatory response, regulation of cell growth and proliferation, cell cycle activation, stress fiber reorganization and remodeling of the extracellular membrane, and change to a motile phenotype. Regulation of cell survival, down-regulation of cell cycle progression, and suppression of inflammatory response characterize the late stage at 96 hours.

The network pathways at the three studied time points of EMT highlight the expected relationships between differentially expressed genes and include both time-dependent genes and those modulated throughout the process (Figure S2). Although these interactive maps are particularly useful for interrogating signals within a single network, they become quite complex when multifaceted cell remodeling takes place. This complexity for EMT in the first 6 hours is very obvious and gradually attenuates until only a few interacting pathways are operative at 96 hours. These pathway diagrams suggest transcriptional control of EMT can be understood as a set of interdependent and changing networks.

### CREs as probes for EMT networks

As highly conserved regulatory elements dominate morphological changes during embryological development [44], [45], [46], [47], we also probed various transcriptional relationships between EMT transcription factors and their binding to *cis*-response elements (*CREs*) among involved promoters. We recovered the *CREs* of all up or down-regulated genes and compiled the most frequent *CREs* at each time point of EMT using TRANSFAC [40], the largest repository of experimentally validated transcription factor binding sites.

The total number of the *CREs* was plotted as a percentage of their frequency among the total number of genes at a particular time point (Table S1 and Figure S3). As up-regulated genes are the majority of all changed genes, the most frequent *CREs* among all up-regulated genes are shown in Figure S3A, those of up-regulated transcription factors (Figure S3B), and the most frequent *CREs* among down-regulated genes (Figure S3C); the down-regulated transcription factors represent a small number of all factors (*Scand1*, *Taf6l*, *Tsc22d3*, *Zipro1*, *Hoxb1* at 6 hours; *Tcf2* at 18 hours, and; *Irf8*, *Lmcd1*, and *Pax8* at 96 hours) and the most frequent *CREs* in the totality of up-regulated genes are similar to those of up-regulated transcription factors. The percentage number in the plots exceeds 100% as some *CREs* are repeated more than once, forming clusters among promoter sequences. The results suggest that distribution of the most frequent *CREs* at any given time point is non-random.

The most frequent *CREs* among all up-regulated genes are *SRY*, *SP1*, *Evi-1*, *GC-box*, *Elk-1*, and *FTS-1*. *FTS-1* is already recognized as a master regulatory element in EMT [15]. Interestingly, the percentage of *SRY*, and to a lesser extent *SP1*, is highest in the genes up-regulated at the onset of EMT and then both decline rapidly, while the percentages of *FTS-1* remain active throughout, *GC-box*, *Elk-1* and *Evi-1* decline by 35–60%. The contribution of *RREB1* and *GC-box* among transcription factors, however, increases with EMT over time. This pattern of differential representation over time is similar in the up-regulated transcription factors (Figure S3B) with the exception of *GC-box*, which increases in percentage from 18 to 96 hours. The overall similarity in frequency of *CREs* between all genes and the cluster of up-regulated transcription factors suggests a common pattern for the functional clusters in EMT.

A similar trend of time-dependent differential representation for the most frequent *CREs* was found in down-regulated genes, where the most frequent *CREs* are *CREB*, *RREB1*, *USF*, *GC-Box*, and *SP1* (Figure S3C). It is interesting that both *GC-Box*, and *SP1*, which peak at 18 hours, are also listed as most frequent of the up-regulated genes. This finding may reflect their propensity to partner with multiple co-factors that modulate transcription as heterologous complexes.

### Knockdown of transcription factors regulated by high frequency CREs

**Figure 3:**
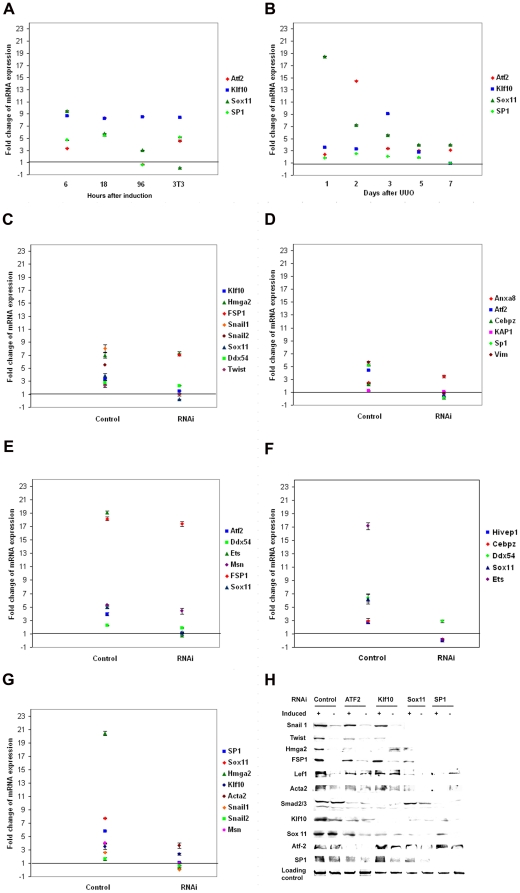
siRNA inhibition of the four up-regulated in EMT transcription factors alters the expression of the marker genes. (**A**) Expression levels of Klf10, Atf2, Sox11, and SP1 in EMT and (**B**) during UUO-induced fibrogenesis. Effects of their RNAi on the expression of EMT markers having the corresponding *CREs* (**C–G**, qRT-PCR; H, Western blot). The mean results from three independent replicates are expressed as fold change compared to the expression in naïve MCT cells (**A**), contralateral kidney (**B**), or specificity control (**C–G**). **C** and **D**. RNAi of Klf10 modulates the expression of most of the EMT marker genes. (**E**–**G**). Modulation of the expression of the marker genes under RNAi of Atf2 (**E**), Sox11 (**F**), and SP1 (**G**). (**H)**. Expression of the EMT markers at the protein level. The loading controls for the western blot represent Coomassie staining of replicate gels.

The finding that particular *CREs* prevail at different stages of transition with a statistically significant distribution at given time points raises the question of how these changes relate to the differential regulation of the EMT transcriptome. The transcription factors up-regulated in EMT are displayed in Table S3 together with their most frequent *CREs*. Underlined are four high CRE frequency transcription factors that were chosen for further study for their relevance to TGF-β-induced EMT (*Atf2* [48], [49], *SP1* [50], [51], [52], *Klf10* [53], [54], and *Sox11* [55]). All four transcription factors are induced within 6 hours and remain up-regulated at 18 hours with two, *Klf10* and *Sox11*, being up-regulated throughout EMT. The basal levels of expression of the four transcription factors are also higher in control fibroblasts compared to non-induced tubular epithelial cells (Figure 3A). These four transcription factors are also up-regulated to varying extent in fibrotic kidney (Figure 3B).

**Figure 4:**
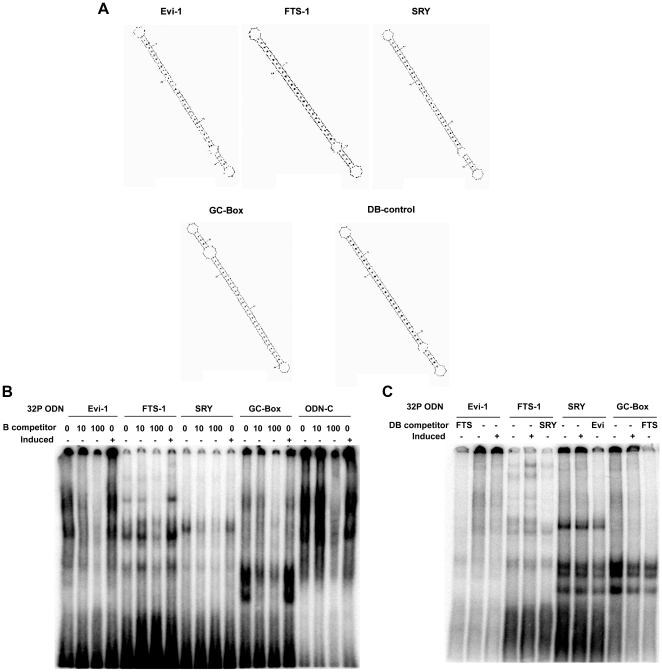
Oligodeoxynucleotide decoys (ODN) compete transcription factors for binding to their respective *CREs* and inhibit formation of complexes between *CREs* and nuclear protein. (**A**) Structure of the ligated at both ends decoys (dumbbells). DB-control- random oligodeoxynucleotide used as a negative control. (**B**) and (**C**). EMSA of radiolabelled *CREs* forming complexes with nuclear protein from induced (+) or non-induced cells (−) that are competed by their respective decoys (**B**) or heterologously by others (**C**).

We next studied the effect of RNA inhibition (siRNA) of these four transcription factors on the expression of the EMT marker genes described earlier. Epithelial cells were transfected with the appropriate target siRNA or a specificity control, and a day later were induced to EMT for 6 hours. Only the levels of expression of EMT marker genes having binding sites for the corresponding transcription factors in their promoters were monitored (Figure 4C–G). The targeted inhibition of each of the four selected transcription factors suppresses the expression of corresponding genes to different degrees. *Klf10* binding sites are represented in most of the genes studied and its inhibition negatively affects the expression of the selected EMT markers as well as the other three factors under study, *Sox11*, *Atf2*, and *SP1* (Figure 3C and 3D). The inhibition of *Sox11*, *Atf2*, and *SP1* also negatively affects the expression of genes having their corresponding binding sites (Figure 3E–G).

Alternatively, the expression of *FSP1*, *vimentin*, and *Hmga2*, having *Klf10* binding sites, are not affected by its inhibition. *FSP1* has binding sites for *Atf2* as well, but again is not affected by its inhibition (Figure 3E). As was pointed out earlier [15], the transcriptional complex, *CBF-A/KAP1/FTS-1*, seems to effectively regulate *FSP1* activation alone. The expression at the protein level generally follows changes in the corresponding levels of mRNA with some exceptions (Figure 3I). EMT marker genes that do not have binding sites for some *CREs* are down-regulated by their inhibition, for example *Snail1* and *Sox11*, *Hmga2* and *Atf2*, *FSP1* and *SP1*. This apparent disparity cannot be explained solely by differences in the protein half-life because it also exists at the level of transcription and thus implies the existence of overlapping *CRE* networks. Despite their pronounced effect on the expression of the EMT marker genes, knockdown of any of the four transcription factors postpones transition to fibroblast phenotype by 5–6 days (data not shown), but does not abrogate EMT.

### Knockdown of high frequency *CREs* leads to apoptosis and attenuation of EMT

The finding that knockdown of each of the four above transcription factors modulates target EMT genes and slows down phenotypic transition suggests the most frequent *CREs* contribute to a highly regulated transcription network.

**Figure 5:**
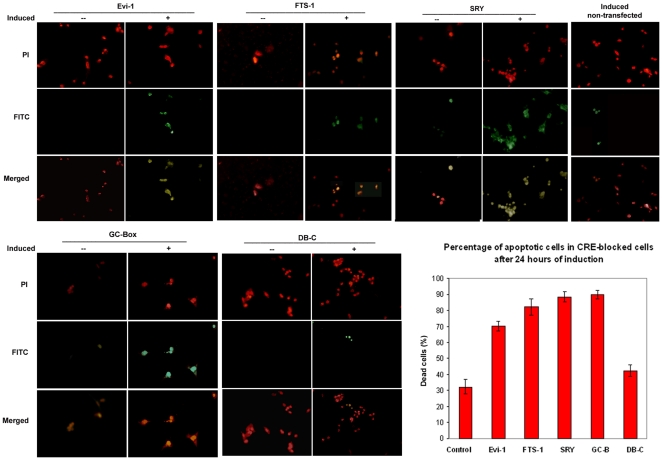
Inhibition of the most frequent *CREs* by their decoys leads to apoptosis of cells induced to EMT. Induced (+) and non-induced (−) cells respond differently to the effects of the decoys after 24 hours. Apoptosis was evaluated using the TUNEL technology (see Materials and Methods). Magnification 40×. Inset: quantitative evaluation of the apoptotic events. Control: non-transfected, but induced to EMT cells; DB-C: cells transfected with the DB-control decoy.

To interrogate this notion further we blocked each of the four most frequent *CREs* (*SRY*, *Evi-1*, *GC-Box*, and *FTS-1*) among up-regulated genes to establish their effect on the stability of transitional cells during the expression of EMT genes. To this end, we designed decoy oligodeoxynucleotides (ODN) for *SRY*, *Evi-1*, *GC-Box*, and *FTS-1* as well as irrelevant controls to known *CREs* [56], [57], [58]. We hypothesized that this approach would interfere with an entire set of transcription factors and their associated binding-proteins. Each decoy oligonucleotide consists of two repeating *CREs* ligated at both ends for nuclease protection in a dumbbell structure (Figure 5A) [41]. Radiolabeled forms of each of the selected *CREs* generate complexes in EMSA that are more pronounced with nuclear protein from EMT-induced tubular cells (Figure 4B). The activity of the decoys was established by competition using 10 to 100-fold molar excess to challenge complexes formed with different *CRE*s. Competition with heterologous decoys (Figure 4C) established that *FTS-1* competes *Evi-1* and *GC-Box*, while *Evi-1* competes *SRY and SRY* competes *FTS-1* albeit partially.

**Figure 6:**
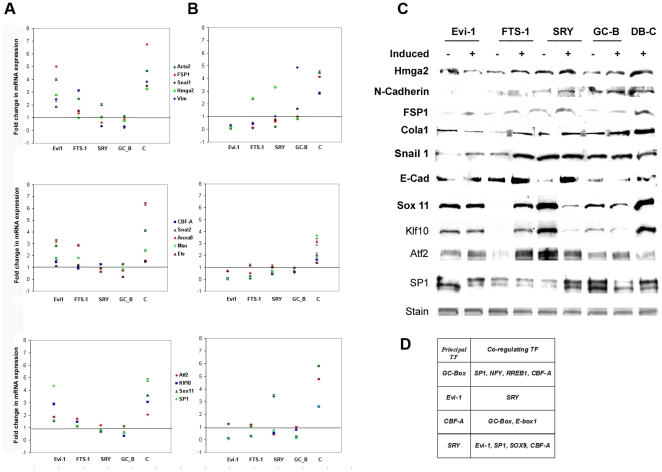
Decoys of the most frequent *CREs* modulate the expression of EMT marker genes. (**A**) after 6 and (**B**) after 18 hours transition. The mean results from three independent replicates are expressed as fold change compared to the expression in non-induced MCT cells. The extent of modulation is specific for each decoy and affects their corresponding *CREs*, but reveals also a heterological type of inhibition, more obvious at the protein level (**C**) that underscores the ubiquitous nature of binding specificity (**D**). The loading controls for the western blots (C) represent Coomassie staining of replicate gels. The transcription factors identified to have specificity to the most frequent CREs in addition to their principal binding sites (**D**) are identified using TFM-Explorer (http://bioinfo.lifl.fr/TFM/TFME/) and rVista (http://rvista.dcode.org/).

EMT normally protects most transitioning cells from apoptosis [46], [59], [60], perhaps through the eventual activation of Snail [61] or Twist [47]. Approximately 10–15% of cells, however, become apoptotic immediately after induction of EMT with TGF-β (Figure 6, induced non-transfected control) in accordance with the known effect of this cytokine on mammalian cells [62]. However, the *CRE* decoys transfected into tubular epithelial cells initiate apoptotic cell death visible after 24 hours of EMT (Figure 5). This substantial apoptotic response is paralleled by modulation in the expression pattern of the EMT markers as measured by qRT-PCR and Western blotting (Figure 6). Neither control decoys in induced cells, nor decoys transfected in non-induced cells engender such a strong apoptotic response.

To test the notion that the most frequent *CRE*s belong to a network we studied the expression of the marker genes in response to all blocked *CRE*s, present or not present on their promoters. As the levels of mRNA encoding the marker genes change during EMT (Figure 2), we monitored their expression at two time points with the highest number of up-regulated genes 6 and 18 hours (Figure 6A and 6B). All four decoys, but not the control, inhibit marker expression to varying extent, which is time- and sequence-specific.

At the onset of EMT (Figure 6A), knockdown of the *GC-Box* has the most pronounced effect on the mRNA levels across the board, replaced by *Evi-1* as a dominant suppressor at 18 hours. Similarly, *SRY*, which is second to *GC-Box* in its inhibition at the onset, is replaced by *FTS-1* later. This finding verifies the dynamic state of transcriptional networks during EMT. Another observation is that inhibition of some *CRE*s affects the expression of genes that do not share that promoter element. This is the case with *Snail2 (Slug)* not having *FTS-1*in its adjacent promoter [14], but being affected by its inhibition (Figure 6A and B). The importance of the *FTS-1* transcription complex for induction and propagation of EMT was reported earlier [14]. We assume that as *FTS-1* also competes *GC-Box* complexes in EMSA (Figure 4C), its inhibition would also affect genes having this *GC-Box* in their promoter. Indeed, the *Snail2 (Slug)* promoter contains this element. Similarly, inhibition of *Evi-1* affects negatively the expression of *CBF-A* and *Hmga2*, genes that do not have this *CRE*, but have *SRY*, which is competed by *Evi-1* in EMSA. This trend of cross-inhibition becomes more obvious at the protein level (Figure 6C) and is most likely caused by formation of heterologous transcription complexes in which at least one of the partners recognizes a specific *CRE*. The propensity of transcription factors to recognize and bind heterologous CREs needs also to be taken into account (Figure 6D).

**Figure 7:**
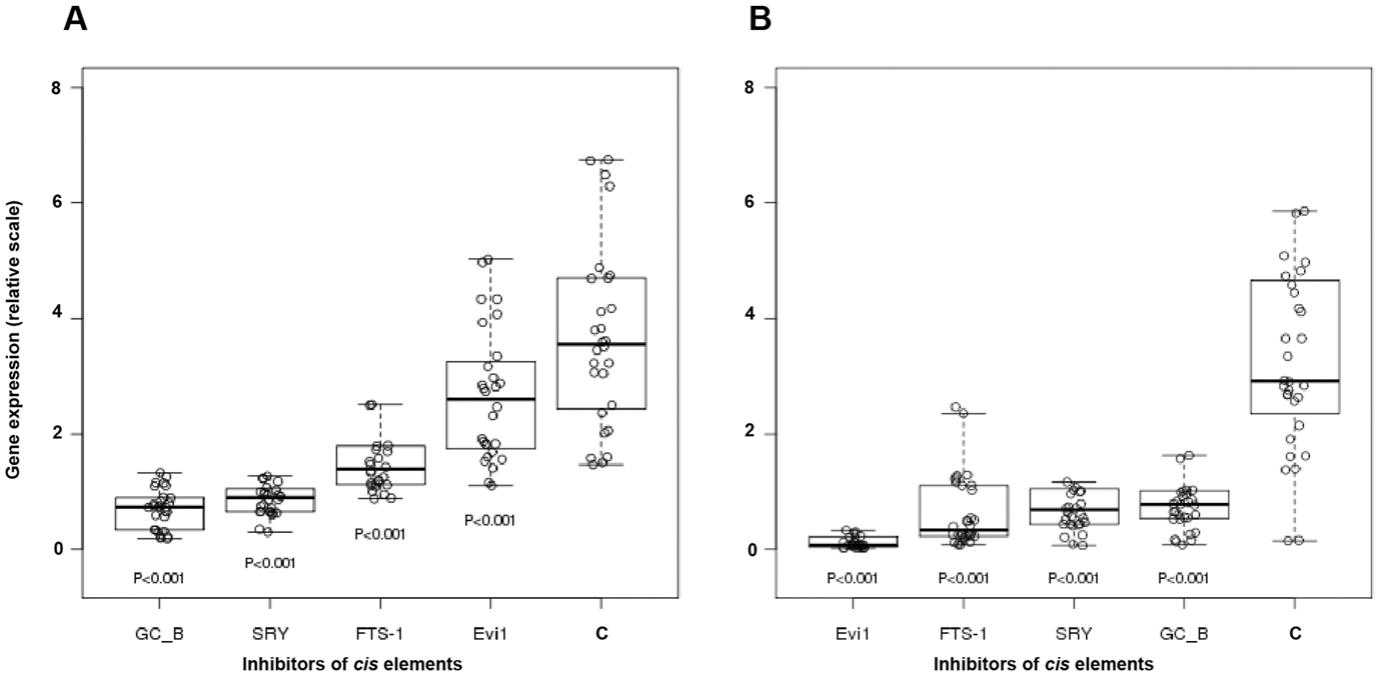
Stage-specific modulation of the expression of EMT markers by inhibition of the most frequent *CREs*. The graph is based on statistical evaluation of the inhibition of the expression of EMT markers and depicts the most effective inhibitory decoys as measured by qRT-PCR for time points 6 hours (**A**) and 18 hours (**B**). Note change of binding preferences with EMT progression. C: DB-control decoy.

**Figure 8:**
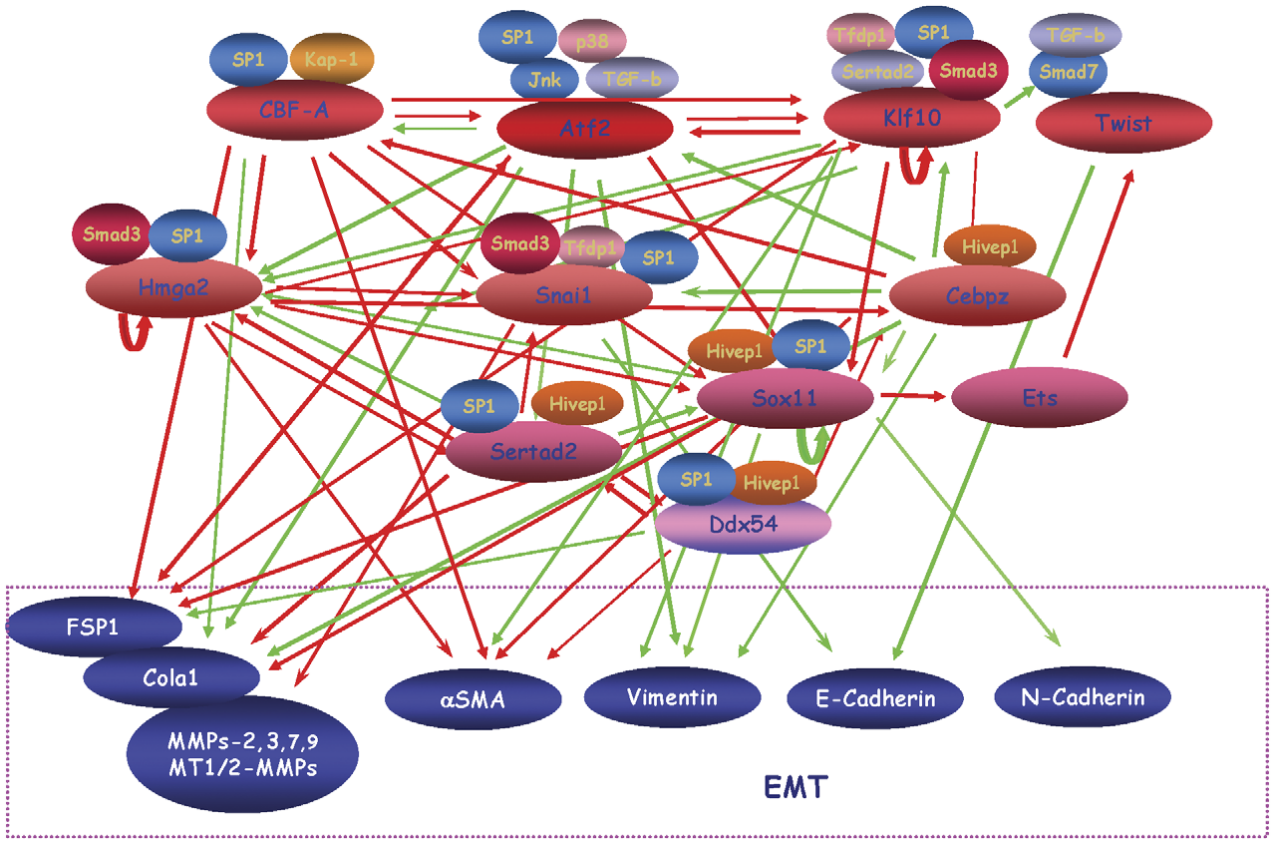
Schematic representation of the complex transcriptional networks in EMT based on the expression of selected EMT markers under RNAi of the four transcription factors. The genes are grouped according to the overall effect on their targets, down- or up-regulation. On top are factors causing the most overall effect, which decreases towards bottom. Red arrows mark upregulating events; green arrows represent downregulation. The density of arrows is a measure of the strength of regulation according to the statistical evaluation. Twist is shown as a reference for its promotional effect on EMT as described elsewhere, although it was not an object in this study.

Based on data collected and statistically evaluated from qRT-PCR at two timepoints of EMT, we compiled a graph expressing the order at which the particular decoys affect the overall expression of EMT markers (Figure 7). Measured by the inhibitory effect on mRNA expression, the order of preference changes with the progression of EMT. Notably, GC-Box that is most efficient at the onset of EMT (A) is rendered most ineffective after 18 hours and is replaced by Evi-1 (B). Taken together, our results imply the existence of a network of transcriptional control that is based on changing the binding preferences of crucial transcription factors at different stages of the transition. Using these data we compiled a table of the most effective *CREs* in the regulation of the EMT markers at two time points, 6 and 18 hours (Table S4). The table lists the most effective decoys in down-regulating the selected markers. Together with the observed variation in competition all data suggest that the four *CREs* are mutually dependent and to some extent, interchangeable. Statistical evaluation of the effects of their inhibition on the mRNA expression of their target genes produced a list of likely hierarchical interrelationships exemplified in Figure 8.

## Discussion

### Regulatory networks in EMT

EMT in adult cells confers the property of movement in once stationary epithelial cells [14], [47], [59] through a variety of transcriptional networks forming fibroblasts [1]. Increased transcriptional gene activity prevails in EMT for at least 18 hours after which it becomes down-regulated by 96 hours. This remodeling of the transcriptome begins before morphological changes are apparent [15]. Variations in the ratio of up-regulated genes during the time course of EMT suggest that key gene clusters are dynamic events with transcription of most up-regulated genes switching on and off during transition, while down-regulated genes are generally silent throughout the transition interval. Approximately 20% of all changed genes in transitioning epithelia are not shared with control fibroblasts, which may reflect the unique transcriptional patterns of parental cells [1] or the axial variability in fibroblast subpopulations [63].

The functional clustering of differentially expressed genes suggests a complex network of regulatory pathways execute cell remodeling as a hierarchy. The signaling networks activated during inducible EMT by TGF-β and EGF [13], [14] are complex and intrinsically involve MAP kinases leading to activation of other transcription factors [64]; EGF action is an important modulator of TGF-β action (in preparation). Our finding that the pattern of expression of *in vitro* transcriptional genes is similar to a classical *in vivo* model of kidney fibrosis validates the results of the DNA microarrays.

### Regulation of gene expression through transcription factor binding sites

The importance of *cis*-regulatory modules for gene regulation as a key component of gene regulatory networks has been recently been stressed [30], [65] and numerous efforts to comprehend the regulatory networks through transcription factor binding sites [28] have been made in relation to development [25], [66], [67] and evolution [44]. By a combination of ChIP assay, expression profiling, and gene knockdown Kubo *et al.* were able to reveal the cis-regulatory networks during embryonic development of amphibians [67].

Mapping the *CREs* of the up-regulated gene clusters at various stages of EMT is evident in Table S3, which lists the up-regulated transcription factors during the first 18 hours and throughout EMT. Their importance was further tested by suppression of four selected transcription factors that contain the most represented *CREs* in their promoters. They were selected also on the basis of their functions as listed in NCBI databank as follows: the activating transcription factor-2 (ATF2), is modulated by TGF-β and operates as a key regulatory molecule in cell growth, Sp1 participates in heterologous complexes with other transcription factors to regulate transcription of a variety of genes. Krüppel-like factor 10 (Klf10) is known to antagonize TGF-β signaling, while Sox11, SRY (sex determining region Y)-box 11 is involved in the regulation of cell fate decisions. The selective inhibition of the four normally up-regulated transcription factors in EMT brings changes in the expression pattern of the EMT markers and slows down the transition. Down-regulation of any of the four transcription factors also affected the expression of genes not having obvious binding sites while genes that have the appropriate CREs were not affected by RNA inhibition of the corresponding transcription factors. The apparent promiscuity in the alternative binding sites for these transcription factors as shown in Figure 6D emphasizes the complexity of the regulation.

### Importance of the most represented *CREs* for initiation and propagation of EMT

The oligonucleotide decoys designed to block the most represented CREs in cells induced to EMT compete with the transcription factors and block their function [68], [69]. Introduction of any of the four decoys into the epithelial cells affects irreversibly their ability to undergo transition in favor of substantial apoptosis. This is a specific effect, as control decoys do not elicit such response. Taking into account that the four *CREs* were selected for inhibition as being most frequent in the promoters of up-regulated transcription factors, it is likely their blockage disrupts the transcriptional networks activated to carry out EMT. This conclusion is supported by data showing considerable down-regulation of the EMT marker genes and their encoded proteins by blockage of any of the four *CREs*. As negative regulation of apoptosis is one of the predominant functions in the early stages of EMT to allow for cell growth and division (Table S2) disruption of such regulatory networks would also negate this function to enable apoptosis.

Another interesting observation is the change in order of representation of the most frequent *CREs* during the transition from 6 to 18 hours (Figure 7). This likely reflects change of preferences for the transcription factors and their complexes involved in propagation of the transition. It is difficult to ascribe each transcription factor to a particular *CRE* as their binding preferences are ubiquitous and vary in the context and timing of various regulatory networks [70]. In addition, transcription factors that do not have specificity for a particular *CRE* may bind as a complex with another factor specific to the site and thus exert a combined effect on expression of a given gene. Such variability may be necessary in complex processes as EMT in which many interdependent regulatory networks with many transcription factors are involved, as exemplified by Figure S2.

The molecular program of EMT involves intricate networks of dynamic signaling that change from induction to progression until transition reaches a steady state. The complexity of the regulation involved in EMT is highlighted by the interchanging preferences of the transcription factors to *CRE*s and presents a challenge to understanding the role of given transcription factor in a particular network. For this reason monitoring the most frequent binding sites of the transcription factors involved in a particular process offers an alternative approach to comprehend complex regulatory networks. Our results imply the existence of a transcription regulatory network with interdependent regulation through variations of binding preferences to selected *CREs* and by the propensity to form heterologous transcription factor complexes. Like many other networks [61], the regulatory networks in EMT are most likely executed by dynamic and context-dependent formation of transcription admixtures targeting interchangeable *CREs*.

In an effort to unravel the transcriptional regulatory network in EMT, we applied an integrative approach combining DNA microarray with computational biology that generates a comprehensive view of the regulatory interactions at a genome-wide scale. This approach has lead to the creation of new web-based tools [71].

## Supporting Information

Figure S1 **Functional clustering in EMT at the various stages studied.** Genes are represented by their symbols on the right and by accession numbers (left). Up-regulated: red, down-regulated: green and unchanged: black. Some genes with multiple functions participate in more than one cluster.
(TIF)

Figure S2 **Regulatory pathway diagrams of differentially expressed genes at the three EMT stages studied.** Red: up-regulated, green: down-regulated. Note the complexity of the interacting networks at the 6 hour that become more simplistic as the transition progresses.
(TIF)

Figure S3 **Most frequent** ***cis*** **elements in the promoters of all changed during EMT genes at the three timepoints studied.** The total counts of respective *cis*-elements are shown as percentile of their frequency in the total number of genes selected at that time point. (**A**). All up-regulated. (**B**). Transcription factors up-regulated during the transition. (**C**). All down-regulated. Note the changes in frequency of *CREs* with EMT progression. (**D**). Most frequent CREs of the four transcription factors selected for further study.
(TIF)

Table S1 **Summary of differentially expressed genes.**
(DOC)

Table S2 **Major regulatory networks in EMT.**
(DOC)

Table S3 **Transcription factors upregulated in EMT and their most repeated** ***CRE*** **s. Early and intermediate stages (6–18 hours) and throughout the transition (6–96 hours).**
(DOC)

Table S4 **Regulation of the EMT marker genes by most frequent CREs.**
(DOC)

Methods S1 **Supplemental methods.**
(DOCX)
