# Supplementary figures and images for: Transcriptional Networks in Epithelial-Mesenchymal Transition
Source: PLoS One. 2011 Sep 30;6(9):e25354. doi: 10.1371/journal.pone.0025354 (PMC3184133; doi:10.1371/journal.pone.0025354)

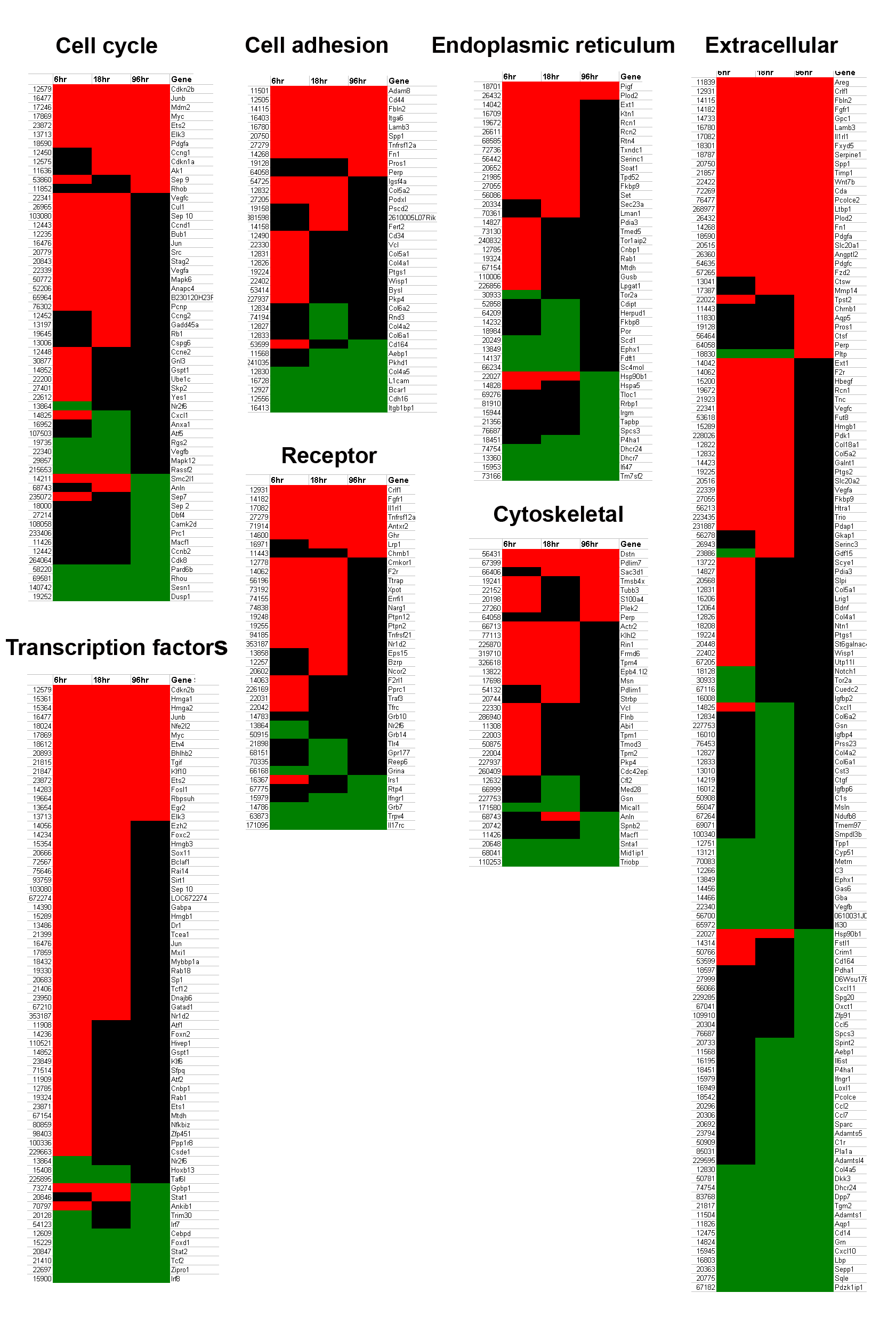

Supplement: Figure S1 — Functional clustering in EMT at the various stages studied. Genes are represented by their symbols on the right and by accession numbers (left). Up-regulated: red, down-regulated: green and unchanged: black. Some genes with multiple functions participate in more than one cluster. (TIF) [file pone.0025354.s001.tif]

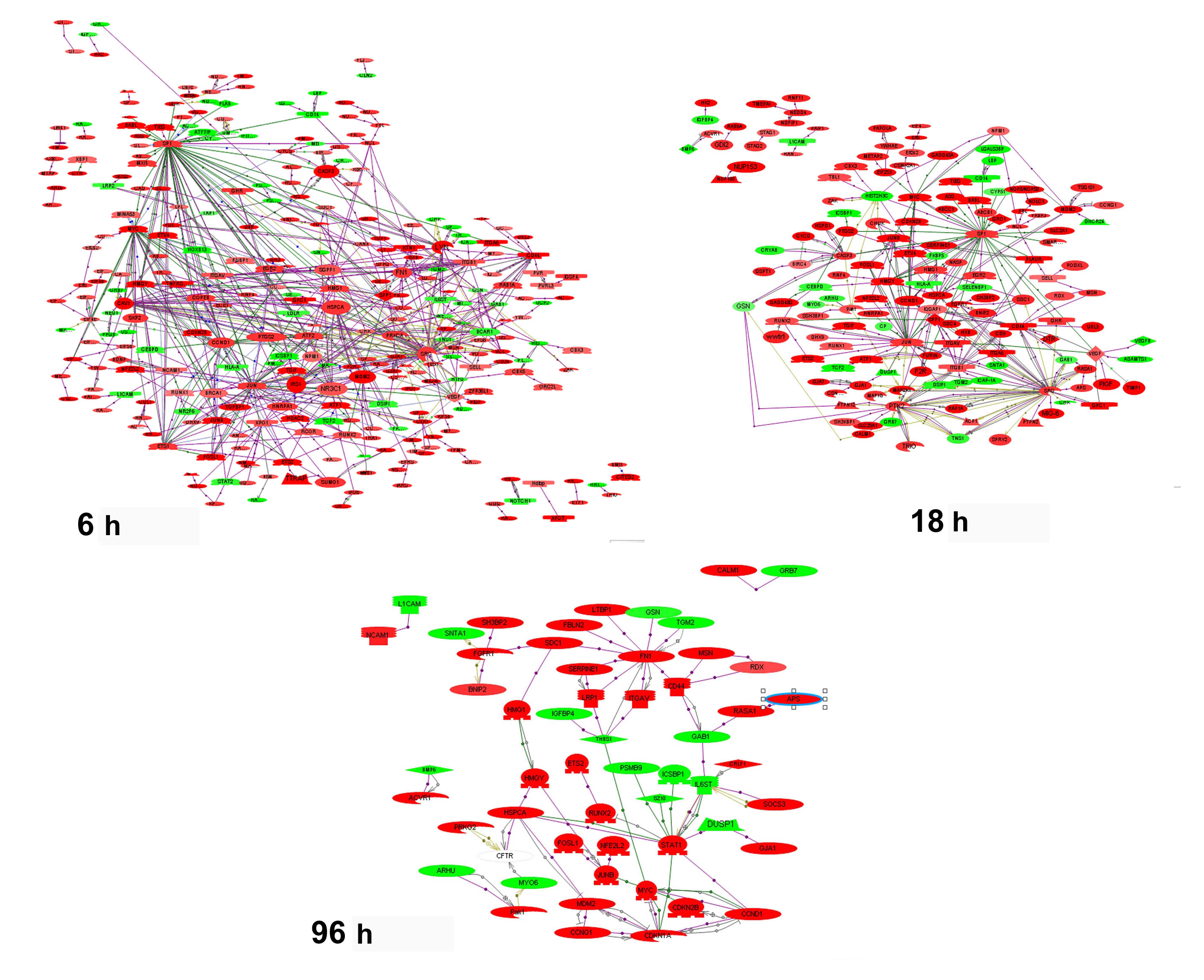

Supplement: Figure S2 — Regulatory pathway diagrams of differentially expressed genes at the three EMT stages studied. Red: up-regulated, green: down-regulated. Note the complexity of the interacting networks at the 6 hour that become more simplistic as the transition progresses. (TIF) [file pone.0025354.s002.tif]

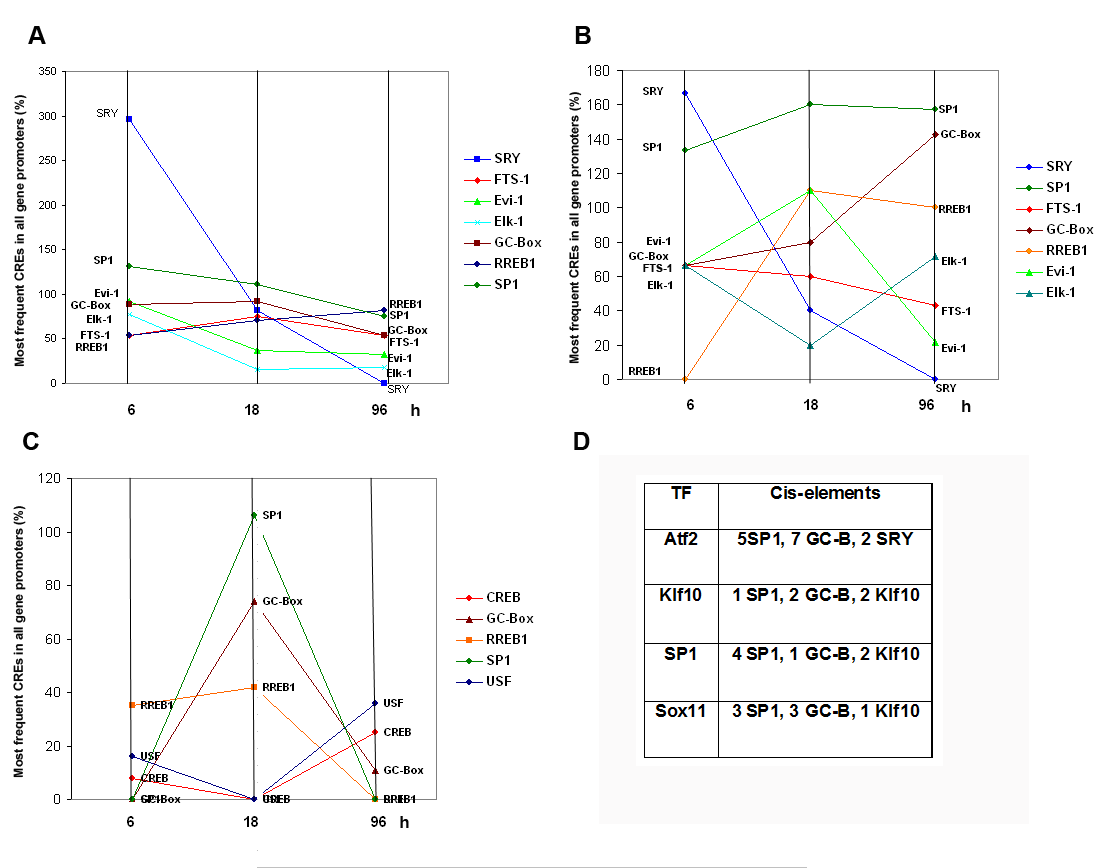

Supplement: Figure S3 — Most frequent cis elements in the promoters of all changed during EMT genes at the three timepoints studied. The total counts of respective cis-elements are shown as percentile of their frequency in the total number of genes selected at that time point. (A). All up-regulated. (B). Transcription factors up-regulated during the transition. (C). All down-regulated. Note the changes in frequency of CREs with EMT progression. (D). Most frequent CREs of the four transcription factors selected for further study. (TIF) [file pone.0025354.s003.tif]
