# Supplementary material for: Transcriptional Networks in Epithelial-Mesenchymal Transition
Source: PLoS One. 2011 Sep 30;6(9):e25354. doi: 10.1371/journal.pone.0025354 (PMC3184133; doi:10.1371/journal.pone.0025354)
Supplement: Table S1 — Summary of differentially expressed genes. (DOC) [file pone.0025354.s004.doc]

| **Time (h)** | **Up** | | **Down** | | **Total** | |
| --- | --- | --- | --- | --- | --- | --- |
|  | **Number** | **%** | **Number** | **%** | **Number** | **%** |
| **6** | **580** | **72** | **226** | **28** | **806** | **42** |
| **18** | **473** | **63** | **278** | **37** | **751** | **38** |
| **96** | **138** | **35** | **256** | **65** | **394** | **20** |
| **Total** | **1,191** | **61** | **760** | **39** | **1,951** | **100** |

**Table S1. Summary of differentially expressed genes**
