# Supplementary material for: Transcriptional Networks in Epithelial-Mesenchymal Transition
Source: PLoS One. 2011 Sep 30;6(9):e25354. doi: 10.1371/journal.pone.0025354 (PMC3184133; doi:10.1371/journal.pone.0025354)
Supplement: Table S2 — Major regulatory networks in EMT. (DOC) [file pone.0025354.s005.doc]

**Table S2. Major regulatory networks in EMT**

| **Clusters** | **Stages of EMT** |
| --- | --- |
|  | **Early Intermediate Late** |
| **Transcription factor** | **regulation of cell growth regulation of proliferation suppression of**  **and proliferation and apoptosis inflammatory**  **response** |
| **Cell cycle** | **activation of cell growth support of cell cycle cell survival and**  **and proliferation, negative activation growth arrest**  **regulation of apoptosis** |
| **Cell adhesion** | **focal adhesion and change to motile regulation of cell- remodeling of ECM phenotype cell adhesion**  **and viabilty** |
| **Cytoskeleton** | **stress fiber organization actin remodeling intracellular signaling and signaling and**  **regulation of focal actin**  **adhesion reorganization** |
| **Receptor** | **proinflammatory cellular activation of cell downregulation of response proliferation and cell cycle**  **migration progression** |
| **Endoplasmic reticulum** | **regulation of transcription cellular transformation, suppression of**  **and phosphorylation, suppression of lipid cellular response**  **reorganization of the biosynthesis catalytic**  **metabolic pathways activity** |
| **Extracellular** | **modulation of activation of stress upregulation of cell**  **inflammatory response response, cell cycle proliferation and**  **and activation of cell progression and cell signaling,**  **signaling towards cell migration; mesenchymal**  **migration and signal differentiation, cell**  **morphogenesis transduction, suppression signaling, organ**  **of stress- and morphogenesis,**  **pro-inflammatory downregulation of**  **response immune and**  **cellular**  **response** |
