# Supplementary material for: Transcriptional Networks in Epithelial-Mesenchymal Transition
Source: PLoS One. 2011 Sep 30;6(9):e25354. doi: 10.1371/journal.pone.0025354 (PMC3184133; doi:10.1371/journal.pone.0025354)
Supplement: Table S3 — Transcription factors upregulated in EMT and their most repeated CRE s. Early and intermediate stages (6–18 hours) and throughout the transition (6–96 hours). (DOC) [file pone.0025354.s006.doc]

**Table S3. Upregulated in EMT transcription factors and their most frequent *CRE*s**

**Onset (6 hours) and transition to 18 hours**

**ID Gene Most common *cis* elements**

***12607 Cebpz Elk1, Evi1, SRY, SP1, ISRE***

***58172 Sertad2 SP1, SOX9, Evi1, TATAB***

***110521Hivep1 SRY, Evi1, Meis1, FTS-1, SOX9***

***71990 Ddx54 SP1, GC-BOX, ISRE, FTS-1, SRY***

***22433 Xbp1 SRY, SOX9, SP1, FTS-1, ISRE***

***18813 Pa2g4 SRY, SOX9, FTS-1***

***97064 Wwtr1 FTS-1, SP1, GC-BOX***

***21399 Tcea1 SP1, FTS-1, Egr3, NF-Y***

***17684 Cited2 SP1, GC-BOX, SRY, Klf10***

***14390 Gabpa USF, SRY, Klf10***

***13486 Dr1 SP1, GC-BOX, FTS-1***

***14056 Ezh2 E2, RREB-1, FTS-1, SRY***

***14234 Foxc2 AP4, FTS-1, Egr2, RREB-1***

***72567 Bclaf1 SP1, SRY, GC-BOX, Tgif, FTS-1***

***13017 Ctbp2 SP1, RREB-1, Egr2, Lef1***

***20683 Sp1 SP1, GC-BOX, Klf 10***

***11909 Atf2 GC-BOX, SP1, SRY, c-MYC***

**Upregulated throughout EMT (6 to 96 hours)**

**ID Gene Most common *cis*-elements**

***16477 Jun-B GC-BOX, SP1, v-MYB***

***17869 Myc GC-BOX, SP1, v-MYB, FTS-1***

***13713 Elk3 SP1, GC-BOX, NGFI-C, FTS-1,***

***20893 Bhlhe40 GC-BOX, SP1, v-MYB, MYOD***

***21807 Tsc22d1 SP1, FTS-1, NF-Y***

***18024 Nfe2l2 GC-BOX, MYOD, SP1, v-MYB***

***20666 Sox11 GC-BOX, SP1, Klf10***

***15361 Hmga1 GC-BOX, v-MYB, MYOD***

***21847 Klf10 GC-BOX, SP1, Klf10, v-MYB,***

***15364 Hmga2 GC-BOX, SP1, v-MYB, FTS-1***

***14283 Fosl1 SP1, GC-BOX, NF-Y***

***23872 Ets2 SP1, GC-BOX, FTS-1, NF-Y***

***21815 Tgif1 GC-BOX, SP1, MYOD, FTS-1***

***18612 Etv4 v-MYB, GC-BOX, FTS-1, SP1***
