# Supplementary material for: Transcriptional Networks in Epithelial-Mesenchymal Transition
Source: PLoS One. 2011 Sep 30;6(9):e25354. doi: 10.1371/journal.pone.0025354 (PMC3184133; doi:10.1371/journal.pone.0025354)
Supplement: Table S4 — Regulation of the EMT marker genes by most frequent CREs. (DOC) [file pone.0025354.s007.doc]

**Table S4. Transcriptional regulation of EMT markers by the most frequent CREs. Statistical significance: p<0.0001**

| **Gene** |  | | | |
| --- | --- | --- | --- | --- |
|  | **Most effective** | | **Less effective by order of magnitude** | |
|  | **6 h** | **18 h** | **6 h** | **18 h** |
| ***Annex 8*** | ***SRY= GC-B*** | ***Evi-1*** | ***Evi-1=FTS-1*** | ***FTS-1=GC-B>SRY*** |
| ***Atf2*** | ***GC-B*** | ***Evi-1*** | ***SRY>FTS-1=Evi-1*** | ***SRY>GC-B>FTS-1*** |
| ***CBF-A*** | ***FTS-1= GC-B*** | ***Evi-1*** | ***SRY=Evi-1*** | ***Evi-1>FTS-1>SRY>GC-B*** |
| ***E-cad*** | ***No change*** | ***Evi-1 = SRY*** | ***No change*** | ***GC-B=FTS-1*** |
| ***Ets*** | ***GC-B*** | ***GC-B = Evi-1*** | ***SRY>Fts-1=Evi-1*** | ***SRY>FTS-1*** |
| ***FSP1*** | ***SRY = FTS-1*** | ***FTS-1 = Evi-1*** | ***GC-B>Evi-1*** | ***GC-B>Evi-1>SRY>FTS-1*** |
| ***HmgA2*** | ***GC-B*** | ***Evi-1*** | ***FTS-1=SRY>Evi-1*** | ***GC-B>FTS-1>SRY*** |
| ***Klf10*** | ***GC-B*** | ***Evi-1*** | ***SRY>FTS-1>Evi-1*** | ***FTS-1>SRY> GC-B*** |
| ***Msn*** | ***GC-B = SRY*** | ***Evi-1*** | ***Evi-1=FTS-1*** | ***FTS-1> GC-B=SRY*** |
| ***SP1*** | ***GC-B*** | ***Evi-1 = GC-B = FTS-1*** | ***SRY>FTS-1>Evi-1*** | ***SRY*** |
| ***PDGF*** | **No change** | ***Evi-1*** | **No change** | ***FTS-1>SRY> GC-B*** |
| ***Snail1*** | ***GC-B*** | ***Evi-1*** | ***SRY>FTS-1>Evi-1*** | ***FTS-1>SRY> GC-B*** |
| ***Snail2, Slug*** | ***SRY*** | ***Evi-1*** | ***FTS-1= GC-B>Evi-1*** | ***FTS-1>SRY> GC-B*** |
| ***Sox11*** | ***SRY*** | ***GC-B*** | ***GC-B=FTS-1>Evi-1*** | ***FTS-1=Evi-1>SRY*** |
| ***Vim*** | ***GC-B = SRY*** | ***FTS-1 = Evi-1*** | ***Evi-1>FTS-1*** | ***SRY> GC-B*** |
| ***Acta2*** | ***GC-B*** | ***FTS-1 = Evi-1*** | ***SRY>FTS-1>Evi-1*** | ***SRY> GC-B*** |
