# Supplementary material for: Transcriptional Networks in Epithelial-Mesenchymal Transition
Source: PLoS One. 2011 Sep 30;6(9):e25354. doi: 10.1371/journal.pone.0025354 (PMC3184133; doi:10.1371/journal.pone.0025354)
Supplement: Methods S1 — Supplemental methods. (DOCX) [file pone.0025354.s008.docx]

### Supplemental Methods

**Rank-based differential expression for multiple chip measurements per transcript.**

We assume that after data normalization, the gene expression values are similar for a given probe among replicates.  Let being the log2 normalized expression intensity at the *i*th probe in the control (C) and treatment (T) group, and there are and replicates, respectively.  Vector stands for all the pairwise expression difference between group C and T , where comparisons. We use sign test method to test the null hypothesis that vector comes from a continuous distribution with a zero median, against the alternative hypothesis that the median is not zero.  Given there are probes in gene (probe set) , we define vector , where is the sum of all positive and negative signs in vector , , however, = 0 when the null hypothesis is valid.  We assumed the intensities of multiple probes in a probe set represent independent measurements observed over a same transcript and applied Wilcoxon signed-rank test statistic to test the null hypothesis that the vector S_g_ comes from a continuous and symmetric distribution with a zero median. The *p* values computed from the Wilcoxon signed-rank test statistic were further adjusted using Benjamini and Hochberg methods to control for multiple comparisons. The resulting false discovery rates were used for assessing the statistical significance of differential expression. In our default settings, we use a less stringent *p* value < 0.1 for rejecting a null sign test hypothesis; this allows more probes to be included in the subsequent test. A regular false discovery rate < 0.01 threshold is used to identify genes with differential expression. To further reduce the gene list of interest, we compute the fold change for each probe set using weighted average method over all pairwise fold changes in a probe set. Let being correlation coefficient computed from raw perfect match data and replicate intensities, >= 0.1, and >= 0.1, we define weight at the probe as: , and use the average weight in the paired probe as the weight at theth comparison (). If the null hypothesis is accepted in a sign test, the associated weight is assigned as 1.
